# Computational Design and In Vitro and In Vivo Characterization of an ApoE-Based Synthetic High-Density Lipoprotein for Sepsis Therapy

**DOI:** 10.3390/biom15030397

**Published:** 2025-03-11

**Authors:** Ling Guo, Yaxia Yuan, Fang Zheng, Changguo Zhan, Xiangan Li

**Affiliations:** 1 Saha Cardiovascular Research Center, Department of Physiology, College of Medicine, University of Kentucky, Lexington, KY 40536, USA; 2 Department of Pharmaceutical Sciences, College of Pharmacy, University of Kentucky, Lexington, KY 40536, USA; 3 Molecular Modeling and Biopharmaceutical Center, College of Pharmacy, University of Kentucky, Lexington, KY 40536, USA; 4 Lexington VA Health Care System, Lexington, KY 40502, USA

**Keywords:** sepsis, therapy, HDL, computational design, nanoparticle

## Abstract

**Introduction:** Septic patients have low levels of high-density lipoproteins (HDLs), which is a risk factor. Replenishing HDLs with synthetic HDLs (sHDLs) has shown promise as a therapy for sepsis. This study aimed to develop a computational approach to design and test new types of sHDLs for sepsis treatment. **Methods:** We used a three-step computational approach to design sHDL nanoparticles based on the structure of HDLs and their binding to endotoxins. We tested the efficacy of these sHDLs in two sepsis mouse models—cecal ligation and puncture (CLP)-induced and *P. aeruginosa*-induced sepsis models—and assessed their impact on inflammatory signaling in cells. **Results:** We designed four sHDL nanoparticles: two based on the ApoA-I sequence (YGZL1 and YGZL2) and two based on the ApoE sequence (YGZL3 and YGZL4). We demonstrated that an ApoE-based sHDL nanoparticle, YGZL3, provides effective protection against CLP- and *P. aeruginosa*-induced sepsis. The sHDLs effectively suppressed inflammatory signaling in HEK-blue or RAW264 cells. **Conclusions:** Unlike earlier approaches, we developed a new approach that employs computational simulations to design a new type of sHDL based on HDL’s structure and function. We found that YGZL3, an ApoE sequence-based sHDL, provides effective protection against sepsis in two mouse models.

## 1. Introduction

Sepsis affects nearly 19 million people globally each year, with a mortality rate exceeding 30% due to the lack of effective therapies [1,2,3]. Despite over 100 clinical trials aimed at targeting various components of the inflammatory and coagulation pathways, patient survival rates have seen little improvement [4]. Sepsis results from a cascade of dysregulated host responses involving multiple steps: Upon infection, bacteria release endotoxins. Endotoxins activate immune effector cells, leading to the production of inflammatory cytokines and chemokines. These inflammatory mediators activate endothelial cells (ECs), causing endothelial dysfunction characterized by vascular leakage, increased leukocyte adhesion, an altered vascular tone, and a shift towards a procoagulant state. Additionally, sepsis induces hemolysis, where broken red blood cells release highly toxic heme, causing cell damage. The elevated levels of inflammatory cytokines and chemokines further contribute to cell damage, releasing damage-associated molecular pattern molecules (DAMPs) that cause a secondary dysregulated immune response [5]. All of these responses culminate in irreversible multi-organ failure and septic death [6,7,8,9]. A significant challenge is that multiple factors and steps contribute to sepsis, and targeting a single regulatory factor or step has shown limited effectiveness. Therefore, targeting an endogenous factor with multi-protective effects against sepsis might be a promising therapeutic approach [10].

High-density lipoproteins (HDLs), a major component of circulating blood [11,12], are well recognized as protective factors against cardiovascular and other chronic inflammatory diseases [12,13]. HDLs play a crucial role in detoxifying endotoxins. Upon infection, Gram-negative bacteria release lipopolysaccharides (LPSs), which bind to TLR4 on immune effector cells. This binding initiates a downstream signaling cascade, activating proinflammatory genes and leading to the production of high levels of cytokines such as TNF-α and IL-6, which results in cell damage [14]. The proinflammatory cytokines can stimulate immune effector cells to generate more cytokines. A body of evidence indicates that HDLs detoxify LPSs through two mechanisms: (i) HDLs neutralize LPSs [15,16,17,18,19,20,21,22,23], most LPSs in circulation exist in an HDL-bound form [22,23], and HDL-LPS binding attenuates LPS-TLR4 interactions [16,24,25,26]. (ii) Recent studies, including ours, suggest that HDLs act together with their receptor, the scavenger receptor BI (SR-BI), to promote LPS clearance [27,28,29]. In vitro, HDLs promote SR-BI-mediated LPS uptake by 4-fold in SR-BI-transfected HEK cells and by 2-fold in primary hepatocytes [27]. In vivo, mice deficient in SR-BI or HDLs display impaired LPS clearance in LPS or sepsis animal models [27,29,30]. These findings suggest that HDLs neutralize LPSs and promote LPS clearance via SR-BI-mediated LPS uptake, which presents a more efficient mechanism for LPS detoxification than simple neutralization by anti-LPS antibodies. Additionally, lipoteichoic acid (LTA), released by Gram-positive bacteria, activates the TLR2/6 pathway to generate high levels of inflammatory cytokines, causing cell damage. Similar to LPSs, LTA is associated with HDLs in circulation and the binding of HDL-LTA neutralizes LTA [31,32]. Given the structural similarity between LPSs and LTA, it is likely that HDLs neutralize LTA and promote LTA clearance via SR-BI-mediated LTA uptake. In addition, HDLs have other activities, such as the suppression of inflammatory signaling in immune effector cells and the inhibition of EC activation [15,16,17,18,19,20,21,22,23,24,25,26,27,28,29,30,31,32,33]. Thus, HDLs may present a promising target for sepsis therapy.

Numerous clinical studies have shown that the levels of HDLs drop markedly in septic patients, and this is associated with a poor prognosis [10,34,35]. We used ApoA-I-null mice as an HDL-deficient model and tested the role of HDLs using cecal ligation and puncture (CLP) as a model of sepsis. We found that a deficiency in HDLs led to a susceptibility to CLP-induced death, as well as less LPS neutralization and LPS clearance [30]. We further found that increasing the HDL levels by overexpressing ApoA-I improved the survival of CLP-induced mice [30]. These clinical and experimental findings strongly suggest that a decrease in HDL levels is a risk factor for sepsis and that raising the circulating HDL levels may provide an efficient therapy for sepsis. A number of earlier studies showed that sHDL treatment improves the survival of LPS-challenged animals [36,37,38,39]. Using a Gram-negative bacterial infection model, Quezado et al. showed that the administration of sHDLs suppressed inflammatory cytokine production, but the sHDLs failed to improve the survival in the study due to the toxicity and impurity of the sHDL product [40]. An earlier study using the ApoA-I mimetic peptide 4F showed that the mimetic peptide 4F treatment increased the HDL cholesterol levels and improved the survival of CLP-treated rats [41]. Unfortunately, the survival was only monitored for two days in that study. We recently utilized an improved sHDL, known as ETC642 (the most potent one of the known sHDL particles) [42,43], and tested its efficacy in CLP-challenged mice. ETC642 is a 22-amino acid ApoA-I mimetic peptide bound to phospholipids to form sHDL nanoparticles. A single dose of ETC642 increased the HDL cholesterol level for up to 48 h in a dose-dependent manner in humans [44,45]. We administered ETC642 to C56BL/6J (B6) mice and found that the ETC642 treatment significantly improved the 7-day survival rate of CLP-treated mice [10]. These studies revealed that the administration of an sHDL could be a potentially effective therapeutic approach, but a more potent sHDL nanoparticle is highly desired as a practically useful therapeutic agent for the treatment of sepsis.

An HDL is a nanodisc made of a dimer of the ApoA-I protein. Thus, the previously reported sHDL nanoparticles were all based on the ApoA-I sequence and designed by simply modifying the ApoA1 sequence. In this study, we designed new sHDLs by a computational approach based on two considerations: (1) Structurally, HDLs are made of a dimer of the ApoA-I protein, which contains several paired positively and negatively charged residues. We speculated that these pairs of charged residues are essential for the formation of a parallel dimer structure as well as the function of HDLs. (2) Functionally, HDLs neutralize endotoxins and regulate endotoxin-induced inflammatory responses. We employed a validated computational approach capable of reliably predicting the binding free energies of ligands like the endotoxins LPS and LTA when interacting with large nanoparticles like sHDLs, and designed a new generation of sHDL nanoparticles that can potently bind with LPSs and LTA. Using the computational approach, we designed four new types of sHDLs, two based on the ApoA-I sequence (YGZL1 and YGZL2) and two based on the ApoE sequence (YGZL3 and YGZL4). Using two mouse models of sepsis, we demonstrate for the first time that an ApoE-based novel type of sHDL nanoparticle, YGZL3, provides effective protection against sepsis.

## 2. Materials and Methods

### 2.1. Computational Details

#### 2.1.1. Coarse-Grained (CG) Model of YGZL Peptides

In constructing the CG model of YGZL peptides, the process began by mimicking the repeated α-helical fragment of ApoA-I. The protein builder function of PyMol [46] was used to construct a standard α-helical secondary structure with the designed amino acid sequence as an initial atomistic model. This atomistic model was then transformed into a CG model, utilizing the MARTINI force field designed specifically for the CG system [47,48].

#### 2.1.2. CG Model of Solvent Molecules, Ions, Sphingomyelin, and 1,2-Dipalmitoyllecithin

The CG model for solvent molecules represented four water molecules as a single CG bead. In the case of ions, they were modeled as charged CG beads, with their first hydration shell being implicitly included [48]. For the lipids, specifically sphingomyelin (PPCS) and 1,2-dipalmitoyllecithin (DPPC), we adopted parameters from the MARTINI force field for lipids [49].

#### 2.1.3. CG Model for the LTA and LPSs

Lipopolysaccharides (LPSs) and lipoteichoic acid (LTA) are complex glycophospholipids with distinct structures that play important roles in sepsis. LPSs consist of three main components: a polysaccharide O-antigen, a core oligosaccharide, and a glycolipid moiety known as lipid A [50]. The lipid A component is critical, as it mediates the proinflammatory and cytotoxic effects of LPSs [51], effectively making it the core moiety of LPSs. On the other hand, the structure of LTA varies among different Gram-positive bacterial species. It typically includes long chains of ribitol or glycerol phosphate, but a glycolipid moiety is a common feature for membrane anchoring [52], serving as the core moiety of LTA. So, we focused solely on the glycolipid moieties of LPSs and LTA, disregarding their more variable components, which allowed for a more streamlined analysis. Parameterization for LTA was based on the parameters of galactosyldiacylglycerol (DGDG) from the MARTINI force field for glycolipids [20,21]. We added an additional Q_a_ bead to represent the extra phosphate group in LTA, with the bond and angle parameters sourced from the phosphatidylinositol (PI) parameters in the same force field. For LPSs, the parameterization process involved iteratively fitting the CG model to the all-atomistic model. This approach aligned with the protocol used in Lopez’s research, ensuring a rigorous and accurate modeling process [53].

#### 2.1.4. Self-Assembly of sHDL Nanoparticles

In the assembly of sHDL nanoparticles, we started by constructing the initial model of the simulation system for YGZL peptides. This involved a random distribution of 20 YGZL peptides, 75 PPCS molecules, and 75 DPPC molecules, following a 1:3.75:3.75 molar ratio, which aligned with the composition of the reported HDL-like ETC642 particle. Each system contained 20 α-helical peptides, equivalent to the number of α-helices in two Apo A-I lipoproteins according to the double-belt model, along with 150 lipids. This composition approximated the reported model of discoidal HDL particles [54,55,56,57]. For a control, a lipid-only particle simulation system was also constructed. This system comprised a random distribution of 75 PPCS molecules and 75 DPPC molecules, without the addition of peptides. Subsequently, both systems were supplemented with CG beads representing water and ions (0.15 M NaCl), creating a physiological environment. The simulations were performed for a total duration of 3 microseconds with a 30 fs integration time step at a temperature of 323 K with an NPT ensemble, using the GROMACS 2016.1 software [58]. This elevated temperature in the CG simulation was chosen to replicate the results of all-atom simulations typically conducted at 300–315 K [12,13,19].

#### 2.1.5. Modeling the Binding Model of sHDL Nanoparticles with LPSs/LTA

To simulate the binding conformation of LTA/LPSs with sHDL particles, our approach involved randomly adding a single molecule of LTA or LPSs into the solvent region of the simulation system, either with the assembled sHDL particle or the lipid-only particle (serving as a control). In this process, any solvent beads overlapping with the LTA/LPS molecules were removed. Additionally, the introduction of the LTA/LPS charge was counterbalanced by adding Na^+^ ions. These systems were then subjected to simulations for a total duration of 1 microsecond each, with a 30 fs integration time step at 323 K, conducted using the GROMACS 2016.1 software.

#### 2.1.6. Calculation of Binding Free Energies of sHDL Nanoparticles with LPSs/LTA

The binding free energy between LTA/LPSs and sHDL particles was estimated through potential of mean force (PMF) calculations. This process was executed by utilizing the pull code and the weighted histogram analysis method (WHAM) [59,60], as implemented in the GROMACS software. The procedure started with the extraction of reaction coordinates from the previously equilibrated LPS/LTA binding simulation systems. This involved pulling the center of mass (COM) of the LPS/LTA molecule 8 nm away from the COM of the sHDL particle across an 80 ns simulation with a 30 fs time step. Following this, 40 sampling windows were set for an additional 10 ns of equilibration with a 30 fs time step and another 10 ns of umbrella sampling at a finer 5 fs time step. These steps were based on the reaction coordinates, spaced at 0.2 nm intervals. To ensure the reliability of the results, the binding free energy of the LPSs/LTA with the lipid-only particle was also estimated, following the same methodological approach for control purposes. Furthermore, to average out any potential fluctuations and enhance the accuracy of the findings, three independent PMF calculations were conducted for each system.

### 2.2. Reagents

The peptide was synthesized by Genscript and the purity was determined to be >95% by an HPLC analysis. Egg sphingomyelin (SM) and 1,2-dipalmitoyl-*sn*-glycero-3-phosphocholine (DPPC) were purchased from Sigma Aldrich (St. Louis, MO, USA). LPSs (*E. coli K12*) were purchased from InvivoGen (San Diego, CA, USA). All other reagents were obtained from commercial suppliers and were of analytical grade or higher.

#### sHDL Preparation

The sHDL nanoparticles were made by co-lyophilization followed by thermal cycling, as described previously [10]. Briefly, the peptide and phospholipids were combined and dissolved in glacial acetic acid or chloroform at a peptide/SM/DPPC ratio of 1:1:1 by weight. The resulting solution underwent rapid freezing in liquid nitrogen, which was followed by freeze-drying overnight to remove the acid. The lyophilized powder was reconstituted in 1X phosphate-buffered saline (PBS) to the desired final peptide concentration and vortexed to completely dissolve it, forming a cloudy white suspension. The resulting solution was subjected to three heat–cool cycles, with each cycle consisting of 10 min of heating at 55 °C and 10 min of cooling at room temperature, at which point a clear solution was formed. The pH of the final sHDL solution was adjusted to 7.4 with NaOH and was then passed through a 0.2 µm sterile filter.

### 2.3. Studies in Animals and In Vitro Analysis

#### 2.3.1. In Vivo Efficacy Analysis

We used two sepsis models in this study: cecal ligation and puncture (CLP)-induced and bacterial infection-induced pneumonia. CLP (21G needle, 2/3 ligation) was performed on 10- to 12-week-old male C57BL/6J mice as described previously [10]. Two hours after the CLP, the mice were treated with 100 µL of PBS or sHDLs at 7.5 mg peptide/kg body weight (i.v.). Their survival was monitored for a 7-day period. Eighteen hours after the CLP, HDLs were isolated from the plasma by sequential ultracentrifugation, as previously described [61,62] (1.5 mL of plasma from five mice was used to make one HDL preparation), and the total HDL cholesterol was measured with a Wako Diagnostics kit. For *P. aeruginosa*-induced pneumonia sepsis, the mice were administered intranasally with 1 × 10^7^ cfu of *P. aeruginosa* in 50 µL of PBS. Two hours later, the mice were treated with/without sHDLs. Their survival was monitored for a 7-day period. The animals were bred at the University of Kentucky’s animal facility. The animals were fed a standard laboratory diet and kept in a 10/14 h light/dark cycle. Mouse tail DNA was used for PCR genotyping. The animal care and experiments were approved by the Institutional Animal Care and Use Committee of the University of Kentucky (protocol title: “Synthetic high density lipoprotein (sHDL) as a therapy for sepsis”; protocol code: 2020-3513; and date of approval: 19 April 2023).

#### 2.3.2. Analysis of NF-κB Expression in HEK-Blue Cells

HEK-Blue cells expressing TLR4 or TLR2 and an NF-κB reporter were used to assess ligand-stimulated NF-κB activation. The cells were cultured to 70% confluency and then treated with LPSs (K12, 1 ng/mL) or LTA (40 ng/mL) in the presence/absence of sHDLs for 16 h. The culture medium (100 μL) was mixed with 100 μL of HEK-Blue Detection, and the activation of the NF-κB reporter was quantified by measuring the absorption at 650 nm.

#### 2.3.3. Analysis of Cytokine Production by RAW264.7 Cells

RAW 264.7 cells were cultured to 80% confluency and then treated with LPSs (K12, 2 ng/mL) in the presence of sHDLs (0, 15, 30, 60, or 120 µg peptide/mL) for 18 h. The concentrations of TNF-α secreted by the cells into the cell culture medium were measured by ELISA.

### 2.4. Statistical Analysis

The data are presented as the means ± SEM or the means ± SD, as indicated in the figure legends. The statistical significance in experiments comparing two groups was determined by a two-tailed Student’s *t*-test. The comparison of more than two groups was evaluated by a one-way ANOVA, which was followed by Tukey’s post hoc analysis. Means were considered statistically significantly different when *p* < 0.05. The survival was analyzed by the log-rank test and Kaplan–Meier plots. The experimental data were statistically evaluated with the GraphPad Prism 9.

## 3. Results

### 3.1. An Efficient Computational Approach to the Design of Novel sHDL Nanoparticles

Concerning our general computational strategy, it is known that amphipathic α-helical proteins, like ApoA-I, have the unique ability to self-assemble around a cylindrical lipid bilayer, forming discoidal lipid–protein particles known as nanodiscs [54,63]. The most widely accepted structural model for nanodiscs proposes that two amphipathic α-helical proteins wrap around the lipid bilayer in a double-belt configuration [57]. This model has gained support from various experimental methods [64,65,66]. Intriguingly, altering the sequence length of the amphipathic α-helical proteins encircling the lipid bilayer can change the size of the nanodiscs without affecting their discoidal shape [54]. Moreover, short α-helical analog peptides derived from the native amphipathic sequence of ApoA-I can also form a discoidal peptide–lipid complex [67], similar in size to nanodiscs composed of native ApoA-I and lipids [68]. This suggests that sHDL nanoparticles, made from short amphipathic α-helical peptides and lipids, are likely to form nanodisc-like structures in an aqueous solution [10]. Our computational design of an sHDL nanoparticle with potentially improved binding affinities for LPSs and/or LTA consisted of three steps (see the Section 2 for the computational details):

(1) Simulate the dynamically stable 3D structures of various sHDL nanoparticles associated with different peptides by performing molecular dynamics (MD) simulations. Notably, for the MD simulation of sHDL nanoparticles, all-atomistic simulations are usually constrained to the nanosecond timescale due to the large system size, which is inadequate for studying nanoparticle self-assembly [69]. Therefore, we opted to use a coarse-grained (CG) model for the MD simulations. The CG model has been effectively used in previous studies to investigate the assembly of lipoprotein particles and permits MD simulations on the microsecond timescale [56,69,70,71], offering a more practical and cost-effective approach for simulating sHDL systems. The same CG model was also employed in our subsequent CG MD simulations mentioned below.
(2) Simulate the dynamically stable sHDL-ligand binding structure by performing the CG MD simulation for each sHDL nanoparticle binding with a ligand (the LPSs or LTA concerned in this study).
(3) Estimate the binding free energy of each ligand with a nanoparticle by performing potential of mean force (PMF) calculations based on the CG MD simulations.

**Figure 1:**
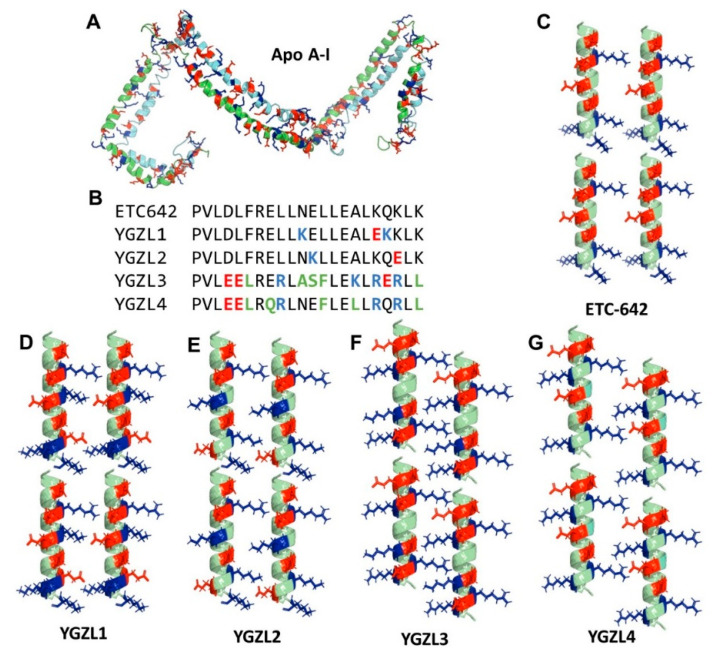
Structures of the designed YGZL peptides in comparison with ApoA-I-based ETC642. The ApoA-I/peptides and key charged residues are shown in cartoon and stick–ball style, respectively. The positively and negatively charged residues are colored in blue and red, respectively. Compared to ETC642, different residues that are neutral are colored green. (**A**) ApoA-I structure. (**B**) Alignment of amino acid sequences of ETC642 and YGZL sHDL peptides. Structures of (**C**) ETC642, (**D**) YGZL1, (**E**) YGZL2, (**F**) YGZL3, and (**G**) YGZL4 in their dimer form. The graphics were generated by PyMOL.

This three-step computational approach enabled us to design dynamically stable 3D structures of various sHDL nanoparticles and predict the binding free energies of LPSs and LTA with various sHDL nanoparticles associated with different peptide choices. Particularly, considering the advantage of our computational approach, our choices of peptides were not limited to the sequence of the Apo A-I protein. As depicted in Figure 1A, the dimer of the Apo A-I protein contains a lot of paired positively and negatively charged residues. We speculated that these pairs of charged residues would be very important for the formation of a parallel dimer structure as well as the function of ApoA-I. However, the ESP-24218 peptide of ETC642 (Figure 1C), derived from a consensus sequence of ApoA-I (Figure 1B), did not thoroughly consider the interaction of charged residue pairs. Therefore, based on the sequence of ESP-24218, we optimized the charged residues to obtain YGZL1 (Figure 1D) and YGZL2 (Figure 1E). Further, we tried to use another apolipoprotein of HDLs, ApoE, to adjust the ESP-24218 more substantially, aiming to explore analogues based on different sequences of ApoA-I and ApoE. After optimizing the matching of charged residue pairs, we designed YGZL3 (Figure 1F) and YGZL4 (Figure 1G) starting from the ApoE sequence.

**Figure 2:**
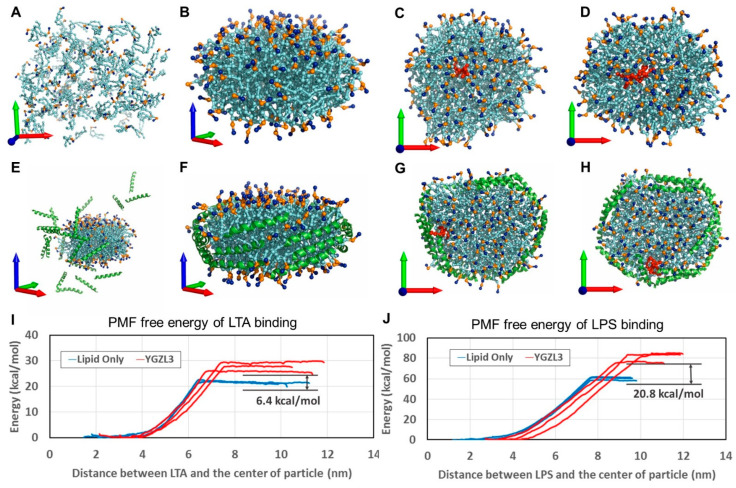
Modeling of sHDL (YGZL3) binding to endotoxin (LPSs or LTA) and the energetics of the binding process. (**A**) The initial state of the lipid-only nanoparticle; all lipids (colored in cyan) are randomly distributed. The positively charged choline head and the negatively charged phosphoric acid group are colored blue and gold, respectively. The X, Y, and Z axes of the coordinate system are shown by red, green, and blue arrows, respectively. (**E**) The initial state of the YGZL3 particle; the peptides are depicted in cartoon style and colored in green. (**B**) The final state of the lipid-only particle; it is self-assembled to a discoid nanodisc in a 3 μs simulation. The hydrophilic surface of the nanodisc is aligned with the XY plane. (**F**) The YGZL3 particle also formed a nanodisc, where all the peptides were arranged around the lipid. (**C**) LTA and (**D**) LPSs are shown in stick–ball style and colored red. They can be inserted into a lipid-only particle. (**G**) LTA and (**H**) LPSs can also be inserted into the YGZL3 nanoparticle; however, they favorably stay near the peptides. (**I**,**J**) Binding free energies of LTA/LPS binding with lipid-only or YGZL3 nanoparticles. The graphics were generated by PyMOL.

Further, the CG MD simulations on these peptide-based sHDL nanoparticles revealed that both YGZL-based sHDL nanoparticles and lipid-only nanoparticles form similar nanodisc structures after self-assembly, as illustrated in Figure 2. In the sHDL structure, the helical peptides are parallelly aligned around the edge of the lipid bilayer. This arrangement is akin to the parallel alignment of amphipathic ApoA-I proteins in the double-belt conformation. This results in the hydrophilic groups on the phospholipids being more tightly packed on both sides of the sHDL nanodisc. In contrast, lipid-only nanoparticles have hydrophilic groups on the phospholipids that are more loosely packed. Although both sHDL and lipid-only nanoparticles captured LPSs/LTA during the self-assembly, the PMF calculations demonstrated increasing binding affinities (with the lower binding free energies) of LPSs and LTA to the YGZL-based sHDL particles, compared to the corresponding lipid-only nanoparticles. Based on the CG MD simulations and the PMF binding free energy calculations, we were able to predict the binding free energies of the sHDL nanoparticles for LPSs and LTA (see Table 1 for the predicted binding free energies). Specifically, according to the computational data, the YGZL3-based sHDL was predicted to be the most promising sHDL with the highest binding affinities (or lowest binding free energies) for both LPSs and LTA.

### 3.2. Targeting HDLs with Synthetic HDLs (sHDLs) for Sepsis Therapy

**Figure 3:**
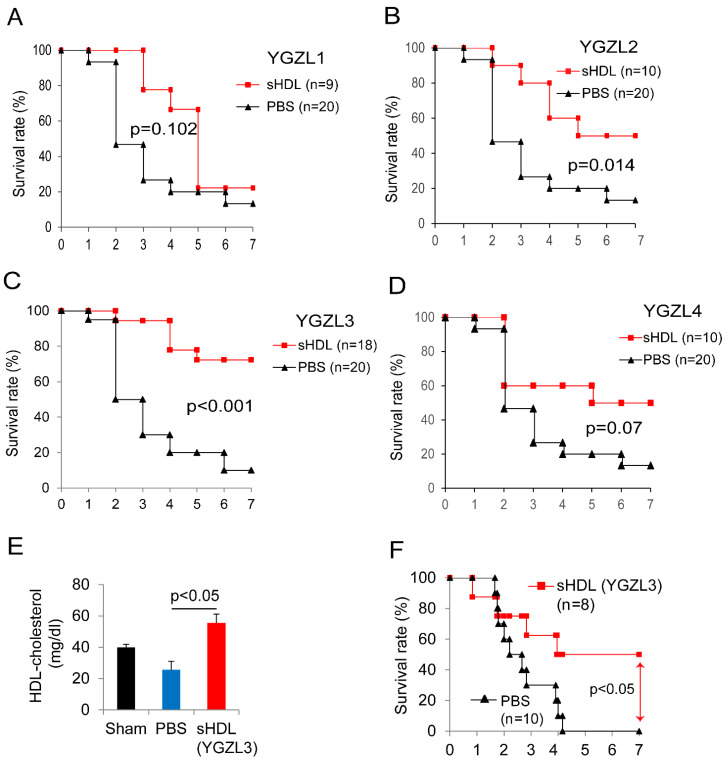
An sHDL treatment restores HDL levels and protects CLP- and bacteria-induced animal death. (**A**–**D**) C57BL/6J mice were subjected to CLP (21G needle, 2/3 ligation) as we described [29,30]. Two hours post-CLP, the mice were treated with different sHDLs at 7.5 mg sHDL protein/kg body weight or PBS (i.v.), and their survival was monitored for 7 days. (**E**) HDL cholesterol concentrations. Plasma was collected at 18 h post-CLP. HDLs were prepared by sequential ultracentrifugation, as described in [62] (1.5 mL plasma from 5 mice was used to make one HDL preparation, n = 3). (**F**) C57BL/6J mice were administered intranasally with 1 × 10^7^ cfu of *P. aeruginosa* in 50 µL of PBS. Two hours later, the mice were treated with sHDLs or PBS and their survival was monitored for 7 days.

Based on a computational prediction, we prepared the sHDL nanoparticles and tested their effects in a CLP-induced sepsis model. To evaluate the therapeutic effect, we treated the septic mice two hours post-CLP. As shown in Figure 3A–D, the sHDLs displayed different protective effects against CLP-induced death. YGZL2 and YGZL3 significantly improved the 7-day survival in CLP-treated mice and YGZL3 showed the best protection. As shown in Figure 3E, the sHDL (YGZL3) treatment increased the plasma HDL cholesterol levels. We also tested sHDLs (YGZL3) in *P. aeruginosa*-induced sepsis—a more clinically relevant bacterial pneumonia sepsis model. The sHDL (YGZL3) treatment significantly protected the mice from *P. aeruginosa*-induced death (50% survival in YGZL3-treated mice compared to 0% survival in PBS-treated mice) (Figure 3F).

### 3.3. sHDLs Suppress Inflammatory Response

**Figure 4:**
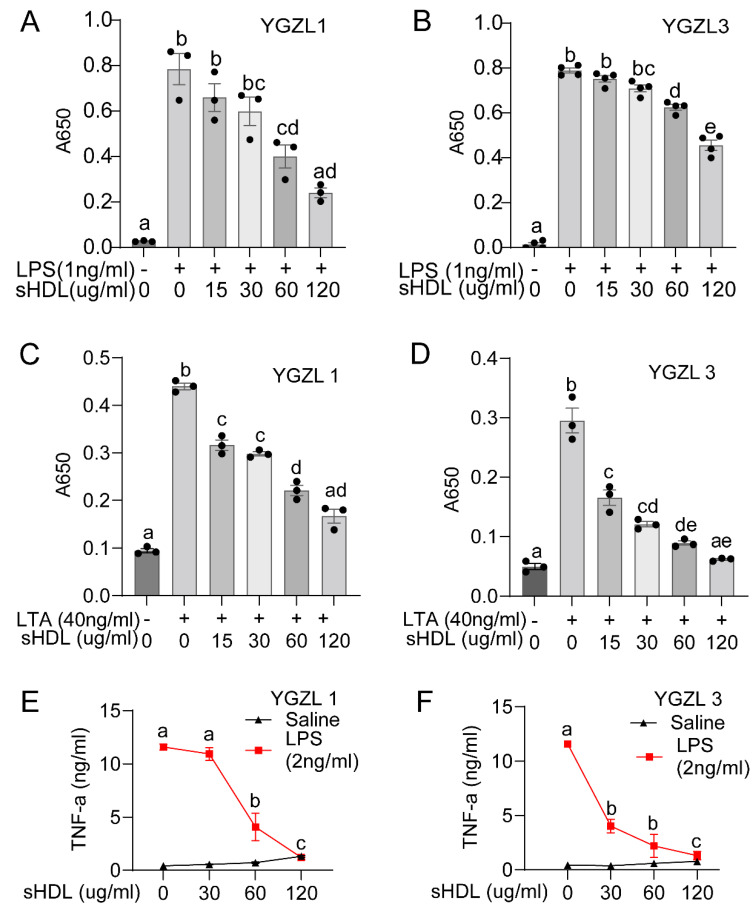
sHDLs suppress the endotoxin-induced inflammatory response. (**A**,**B**) sHDLs suppressed the LPS-induced activation of NF-κB. HEK-Blue cells expressing TLR4 were cultured to 70% confluency and treated with LPSs in the presence of sHDLs for 16 h. Samples (100 μL of the cell culture medium) were mixed with 100 μL of HEK-Blue Detection and the activation of the NF-κB reporter was quantified by measuring the absorption at 650 nm. (**C**,**D**) sHDLs suppressed the LTA-induced activation of NF-κB. HEK-Blue cells expressing TLR2 were cultured to 70% confluency and treated with LTA (**D**) in the presence of sHDLs for 16 h. Samples (100 μL of the cell culture medium) were mixed with 100 μL of HEK-Blue Detection and the activation of the NF-κB reporter was quantified by measuring the absorption at 650 nm. (**E**,**F**) sHDLs suppressed the inflammatory response in RAW cells. RAW 264.7 cells were cultured to 80% confluency and treated with LPSs (K12, 2 ng/mL) in the presence of sHDLs (0, 15, 30, 60, or 120 µg peptide/mL) for 18 h. The concentrations of TNF-α in the medium were measured by ELISA. The means ± SEM are plotted from at least three independent experiments, with each performed in triplicate, and analyzed using a one-way ANOVA. Significant differences (*p* < 0.05) are indicated by different letters; bars with different letters denote significant differences, while bars with the same letter are not statistically different.

The computational design predicted the binding of sHDLs to endotoxins and the regulation of endotoxin-induced inflammation. We validated this by testing the activity of YGZL1 and YGZL3 in regulating the endotoxin-induced inflammatory response in culture cells. YGZL1 and YGZL3 were the least and most effective sHDLs regarding protection against sepsis, respectively. We first investigated the regulation of inflammatory signaling using HEK-Blue cells that were stably transfected to express either human TLR4 or TLR2 with an NF-κB reporter. The cells were challenged with the corresponding receptor ligands (LPS/TLR4, Figure 4A,B; LTA/TLR2, Figure 4C,D) in the presence or absence of various concentrations of YGZL1 or YGZL3. In all cases, we observed a dose-dependent decrease in NF-κB activation with increasing sHDL concentrations. We also tested the effect of sHDLs in microphages (RAW-276 cells). As shown in Figure 4E,F, both YGZL1 and YGZL3 effectively suppressed the TNF-α production induced by LPSs. Of note, YGZL1 and YGZL3 showed different protective abilities against septic death, but both effectively suppressed the endotoxin-induced inflammatory response. This suggests that sHDLs have activity other than regulating the endotoxin-induced inflammatory response. Given the multiple protective activities of HDLs, further studies are required to determine the mechanism underlying the protection against sepsis by YGZL3.

## 4. Discussion

In this study, we developed a validated three-step computational approach to simulate the dynamically stable binding of large nanoparticles, such as sHDLs, with various ligands, including endotoxins. This approach allowed us to predict their binding free energies and design a novel type of more potent sHDL nanoparticles. These nanoparticles can significantly increase the overall plasma HDL levels and effectively suppress sepsis-related inflammatory signaling. Using two mouse models of sepsis, we demonstrated for the first time that an ApoE-based novel type of sHDL nanoparticle provides effective protection against sepsis.

We tested and employed a three-step computational approach to design new sHDL nanoparticles based on our speculation that pairs of charged residues would be important for forming a parallel dimer structure as well as for the function of ApoA-I. Through this new approach, we designed novel types of sHDL nanoparticles (particularly YGZL3) starting from the ApoE sequence. We provided 7-day survival evidence demonstrating that the sHDL YGZL3 protects against sepsis in two clinically relevant sepsis models: CLP-induced and cecal slurry-induced polymicrobial sepsis. The sHDL YGZL3 was administered after the induction of sepsis and not as a preventive measure. Thus, the sHDL YGZL3 may serve as a potentially effective therapy for sepsis.

This research is innovative because, unlike earlier sHDLs made of ApoA-I mimetic peptides, we utilized computer modeling and simulation-based binding free energy predictions to generate a novel type of sHDL based on ApoE—another major component of HDLs. We demonstrated for the first time that an ApoE-based novel sHDL (YGZL3) effectively protected against septic death in two clinically relevant sepsis models. Our computational simulations indicate that the ApoE mimetic peptide bound with phospholipids to form stable nanoparticles, making this sHDL (YGZL3) more effective than the first generation of sHDLs made from the naked peptide.

Additionally, the general concept of the three-step computational approach may be used to simulate the dynamically stable binding of other large nanoparticles with any ligands and predict their binding free energies for the computational design of novel nanoparticles.

## 5. Conclusions

We developed a new approach that employed computational simulations to design a new type of sHDL based on HDL’s structure and function. We found that YGZL3, an ApoE-sequence-based sHDL, provided effective protection against sepsis in two mouse models.

## Figures and Tables

**Table 1:** The PMF binding free energies for YGZL sHDL particles, ETC-642 sHDL nanoparticle, and lipid-only nanoparticle binding with LPSs and LTA.

Particle	Binding Energy (kcal/mol)		Binding Energy Improvement (kcal/mol)	
	LPS	LTA	∆LPS (sHDL—Lipid)	∆LTA (sHDL—Lipid)
Lipid-Only	−59.9	−20.7	N/A	N/A
ETC-642	−77.6	−25.1	−17.7	−4.4
YGZL1 sHDL	−72.2	−23.2	−12.3	−2.5
YGZL2 sHDL	−76.1	−24.4	−16.2	−3.7
YGZL3 sHDL	−80.7	−27.1	−20.8	−6.4
YGZL4 sHDL	−76.2	−25.0	−16.3	−4.3

## Data Availability

All the data associated with this study are available in the main text.
